# Supplementary material for: Placing sensors in sewer networks: A system to pinpoint new cases of coronavirus
Source: PLoS One. 2021 Apr 8;16(4):e0248893. doi: 10.1371/journal.pone.0248893 (PMC8031413; doi:10.1371/journal.pone.0248893)
Supplement: S1 Appendix — (DOCX) [file pone.0248893.s001.docx]

# S1 Appendix - Proofs of Lemmas 1 and 2

**Lemma 1-** In a network with $S$ sensors, $\left| M \right|=k(S+1)$ manholes, *k* a positive integer, and equal Bayesian probabilities of $\rho=1/|M|$, to minimize the expected number of samples, the sensors must be located to have $k$ manholes in each of their respective entry sets. If $\left| M \right|$ is not divisible by *S+1*, then the numbers of manholes in the respective entry sets must sum to $\left| M \right|$ and cannot differ by more than 1.

**Proof of Lemma 1-** Assume that entry sets are equal in size and hold $\frac{\left| M \right|}{S+1}$ manholes each and each manhole has a Bayesian probability of $\frac{1}{\left| M \right|}$; the probably that a given entry set holds the source manhole is $\frac{1}{s+1}$. Then the number of manhole samples according to the Log function is $Q^{\$}=\log(\frac{\left| M \right|}{S+1})$. Assume now that we perturb the solution and move one of the sensors to an adjacent manhole. With this perturbation, we have $S-1$ entry sets with $\frac{\left| M \right|}{S+1}$ manholes, and two perturbed entry sets with $\frac{\left| M \right|}{S+1}+1$ and $\frac{\left| M \right|}{S+1}-1$ manholes. The probability that the source manhole is in each entry set is $\frac{1}{S+1}$ , $\frac{1}{S+1}+\frac{1}{\left| M \right|}$ , and $\frac{1}{S+1}+\frac{1}{\left| M \right|}$ , respectively. Thus the expected number of samples with this perturbation is $Q^{\#}=\frac{s-1}{s+1}\log(\frac{\left| M \right|}{S+1})+(\frac{1}{s+1}+\frac{1}{\left| M \right|})\log\left( \frac{\left| M \right|}{S+1}+1 \right)+(\frac{1}{s+1}-\frac{1}{\left| M \right|})\log(\frac{|M|}{S+1}-1)$, where the three terms are the expected number of samples if the source manhole is in one of the $s-1$ un-perturbed entry sets, or the two perturbed entry sets. Because $Q^{\#}>Q^{\$}$, this perturbation increases the number of samples and worsens the solution.

Thus, in practice, when it may not be possible to have precisely the same number of manholes in each entry set, the best solution is to locate the sensors with approximately equal-size entry sets.

**Lemma 2.** Consider the union set $U_{ab}$ of two manholes $s_{a}$ and $s_{b}$. When the tree of $U_{ab}$ does not have any junctions, the term $Q_{ab}=\log\left( m_{a} \right)p_{a}+\log\left( m_{b} \right)p_{b}$ has a convex hull where $m_{i}$ is the number of manholes in the entry set of sensor $i$and $p_{i}$ is the sum of Bayesian probabilities of the manholes in the entry set of sensor $i$.

**Proof of Lemma 2-** Consider a union entry set $U_{ab}$ that is a tree with no junctions (i.e., a set of manholes consecutively located on a line) that has ${|U}_{ab}|$ manholes located serially next to each other. Recall that we seek to relocate $s_{b}$ in the entry set $U_{ab}$to minimize $Q_{ab}=\log\left( m_{a} \right)p_{a}+\log\left( m_{b} \right)p_{b}$. We index the manholes using $i$ starting from sensor $s_{a}$. Sensor $s_{a}$ is located in manhole $i=1$, the manhole immediately upstream of $s_{a}$ is indexed $i=2$ and the farthest manhole from $s_{a}$ is indexed $i={|U}_{ab}|$. We can now rewrite the term $Q_{ab}\left( i \right)$ as a function of $i$ representing sensor $s_{b}$’s location:

$Q_{ab}\left( i \right)=\log\left( i-1 \right)\sum_{j=1:i-1} \rho_{j}+\log\left( {|U}_{ab} | -i+1 \right) \sum_{j=i:{|U}_{ab}|} \rho_{j}.$ (9)

Assume now that ${|U}_{ab}|\to\infty$. The problem is now converted into continuous space where $x\in(0,\mathcal{M)}$ such that $s_{a}$ is located at point 0 and the length of the junction-free tree is $\mathcal{M}$, which is the distance from $s_{a}$to the farthest node on the tree. We seek the best set of locations, $x^{*}$, within its continuous domain $x\in(0,\mathcal{M)}$. Note that $Q_{ab}\left( x \right)$ in the continuous domain provides a lower-bound for its discrete counterpart $Q_{ab}\left( i \right)$.

Let $F\left( x \right)=\int_{0}^{x} \rho(x)$ where $\rho(x)$ is a probability density function that best represents the discrete probabilities $\rho_{j}$. We can now rewrite (9) as

$Q_{ab}\left( x \right)=\log\left( x \right)F\left( x \right)+\log\left( \mathcal{M-}x \right) \left( 1-F\left( x \right) \right).$ (10)

For a uniformly distributed $\rho\left( x \right)=1/\mathcal{M}$ (which tightens the lower-bound $Q_{ab}$), the second derivative of (10) yields

$$\frac{\partial Q_{ab}\left( x \right)}{\partial x}=\frac{\mathcal{M}+2x}{\mathcal{M}^{2}x\mathcal{-M}x^{2}} ,$$

which is strictly positive because $x\mathcal{<M}$. Thus, $Q_{ab}\left( x \right)$ has a convex hull and because $Q_{ab}\left( x \right)\leq Q_{ab}(i)$, then $Q_{ab}\left( i \right)$ has a convex hull as well.$∎$
