# Supplementary material for: Placing sensors in sewer networks: A system to pinpoint new cases of coronavirus
Source: PLoS One. 2021 Apr 8;16(4):e0248893. doi: 10.1371/journal.pone.0248893 (PMC8031413; doi:10.1371/journal.pone.0248893)
Supplement: Attachment — Submitted filename: manuscript_revision-PONE-D-20-38252_reviewer.docx [file pone.0248893.s002.docx]

Dear Editor

The paper deals with a method for solving sensor placement problem in a sewer system. The Authors formulate the sensor location problem as an integer nonlinear optimization and develop heuristics to solve it. The topic is of high interest for the scientific community. However, the paper often is confused, redundant and wordy (there is possibility to save space for better discussing the application of the proposed framework) and a vigorous revision of the English language is necessary. Furthermore, the paper is not presented in an appropriate fashion (especially the state of art and the discussion of results) and some crucial aspects should be clarified (in particular the methodology). I recommend major revisions be undertaken; listed are comments and suggestions I would like to see addressed before the acceptance of the paper:

The paper lacks a proper state of art about the importance of the monitoring of the water systems, in general, and more specifically of sewer systems and methods already developed for addressing the sensor placement problem, as well as, about the study of the topology of sewer systems. I strongly suggest to improve the Introduction. In this regard, I list some papers could be of your interest to refer to:

- *Banda, T. D., & Kumarasamy, M. (2020). Development of a Universal Water Quality Index (UWQI) for South African river catchments. Water, 12(6), 1534.*
- *Guo, Y., Liu, C., Ye, R., & Duan, Q. (2020). Advances on Water Quality Detection by UV-Vis Spectroscopy. Applied Sciences, 10(19), 6874.*
- *Giudicianni, C., Herrera, M., Di Nardo, A., Greco, R., Creaco, E., & Scala, A. (2020). Topological Placement of Quality Sensors in Water-Distribution Networks without the Recourse to Hydraulic Modeling. Journal of Water Resources Planning and Management, 146(6), 04020030.*
- *Vonach, T., Tscheikner-Gratl, F., Rauch, W., & Kleidorfer, M. (2018). A heuristic method for measurement site selection in sewer systems. Water, 10(2), 122.*
- *Yang, S., Paik, K., McGrath, G. S., Urich, C., Krueger, E., Kumar, P., & Rao, P. S. C. (2017). Functional topology of evolving urban drainage networks. Water Resources Research, 53(11), 8966-8979.*
- *Banik, B. K., Alfonso, L., Di Cristo, C., Leopardi, A., & Mynett, A. (2017). Evaluation of different formulations to optimally locate sensors in sewer systems. Journal of Water Resources Planning and Management, 143(7), 04017026.*
- *Banik, B. K., Di Cristo, C., Leopardi, A., & de Marinis, G. (2017). Illicit intrusion characterization in sewer systems. Urban water journal, 14(4), 416-426.*

In the proposed method there is not reference to the hydraulic conditions of the system; the Authors didn’t carry out hydraulic simulations, neither use specific software for testing the proposed approach. In this regard, I wonder how is it possible to evaluate the efficiency of the method and how reliable are the results. This is a crucial point to discuss for the applicability of the framework in real cases. It seems that the sensor placement problem is addressed without taking into account the physical behaviour of the system that actually is characterised by a daily variability (discharge at each node, dry or rainy conditions, etc…). I strongly recommend to clarify this point;

In order to make the paper more readable and to follow the whole procedure, I suggest to provide a clear general flow-chart of the framework you proposed;

Have you assumed that only a contamination at time can start? Could you discuss all the assumptions behind your method?

Page 16: *“It also allows analyzing the structural properties of the sensor location problem including robustness to perturbations in sensor locations”*. Could you clarify this point and provide more explanations?

What *Q_ab_* stand for? What does it represent?

What is the perspective of the future work? I propose to consider the possibility of completing the discussion on possibilities and limitations of the use of such analysis in other networked systems and infrastructures;

Could you also discuss about the computational complexity of your procedure? The scalability and the time requested for the analysis? This is important for the application to real big-sized systems;

Another crucial point is the fact that there is no explicitly reference to the validation of the proposed approach through performance indices especially because it is not tested on real case study neither compared with methods already developed. I strongly recommend to improve this aspect;

It is not clear the reason why the Authors presented a real case study, reconstruct the sewer network with a procedure and then they applied the sensor placement algorithm to another network. Could you clarify these aspects? Furthermore, why didn't you work directly on the real topology of the case study? All these hypotheses about the system could strongly affect the results?
