# Supplementary material for: Placing sensors in sewer networks: A system to pinpoint new cases of coronavirus
Source: PLoS One. 2021 Apr 8;16(4):e0248893. doi: 10.1371/journal.pone.0248893 (PMC8031413; doi:10.1371/journal.pone.0248893)
Supplement: Attachment — Submitted filename: Comments7 RCL edits.docx [file pone.0248893.s003.docx]

We thank the two reviewers for their constructive comments. According to the reviews, we have made the following major changes in the new draft of the paper:

1- The writing style and English format of the paper are revised according to your recommendations.
2- The literature review section of the paper is extended. We discuss the operational challenges of sampling manholes, the need for installing sensors, and the state-of-the-art of embedded sensors.
3- Section 6 is extended with more analysis on sensitivity to sensor locations.
4- The operational challenges and next steps are discussed in detail in Section 7.

Below please see a detailed response to the comments.

***Reviewer #1: Dear Editor***

***The paper deals with a method for solving sensor placement problem in a sewer system. The Authors formulate the sensor location problem as an integer nonlinear optimization and develop heuristics to solve it. The topic is of high interest for the scientific community. However, the paper often is confused, redundant and wordy (there is possibility to save space for better discussing the application of the proposed framework) and a vigorous revision of the English language is necessary. Furthermore, the paper is not presented in an appropriate fashion (especially the state of art and the discussion of results) and some crucial aspects should be clarified (in particular the methodology). I recommend major revisions be undertaken; listed are comments and suggestions I would like to see addressed before the acceptance of the paper:***

**Response:**

We have revised the writing style of the paper according to your recommendations. Please see the tracked changes version of the submitted manuscript.

***The paper lacks a proper state of art about the importance of the monitoring of the water systems, in general, and more specifically of sewer systems and methods already developed for addressing the sensor placement problem, as well as, about the study of the topology of sewer systems. I strongly suggest to improve the Introduction. In this regard, I list some papers could be of your interest to refer to:***

***- Banda, T. D., & Kumarasamy, M. (2020). Development of a Universal Water Quality Index (UWQI) for South African river catchments. Water, 12(6), 1534.***

***- Guo, Y., Liu, C., Ye, R., & Duan, Q. (2020). Advances on Water Quality Detection by UV-Vis Spectroscopy. Applied Sciences, 10(19), 6874.***

***- Giudicianni, C., Herrera, M., Di Nardo, A., Greco, R., Creaco, E., & Scala, A. (2020). Topological Placement of Quality Sensors in Water-Distribution Networks without the Recourse to Hydraulic Modeling. Journal of Water Resources Planning and Management, 146(6), 04020030.***

***- Vonach, T., Tscheikner-Gratl, F., Rauch, W., & Kleidorfer, M. (2018). A heuristic method for measurement site selection in sewer systems. Water, 10(2), 122.***

***- Yang, S., Paik, K., McGrath, G. S., Urich, C., Krueger, E., Kumar, P., & Rao, P. S. C. (2017). Functional topology of evolving urban drainage networks. Water Resources Research, 53(11), 8966-8979.***

***- Banik, B. K., Alfonso, L., Di Cristo, C., Leopardi, A., & Mynett, A. (2017). Evaluation of different formulations to optimally locate sensors in sewer systems. Journal of Water Resources Planning and Management, 143(7), 04017026.***

***- Banik, B. K., Di Cristo, C., Leopardi, A., & de Marinis, G. (2017). Illicit intrusion characterization in sewer systems. Urban water journal, 14(4), 416-426.***

**Response:**

The literature review section of the paper is extended with a synopsis of most of the above-mentioned papers plus others. We have better positioned our paper within existing literature. In section 2.2 we discuss the need for installing sensors, and in section 2.3 we discuss the state-of-the-art of the embedded sensors.

***In the proposed method there is not reference to the hydraulic conditions of the system; the Authors didn’t carry out hydraulic simulations, neither use specific software for testing the proposed approach. In this regard, I wonder how is it possible to evaluate the efficiency of the method and how reliable are the results. This is a crucial point to discuss for the applicability of the framework in real cases. It seems that the sensor placement problem is addressed without taking into account the physical behaviour of the system that actually is characterised by a daily variability (discharge at each node, dry or rainy conditions, etc…). I strongly recommend to clarify this point;***

**Response:**

Our analysis of the sensor placement problem considers the network topology, and the conservation of wastewater as it flows from the catchment areas to the treatment plant. Given this setting, our analysis is similar in nature to that of the “flow intercept problem” (Berman et al, 1992, 1995), which seeks to choose a set of location in a network to intercept the largest total flow possible. The properties of the wastewater flow fluctuations and of the hydraulic rules are of less concern in our model. We note that the mathematical analysis is often made difficult when modeling the nonlinear system hydraulics within the water system graph. Also, in Giudicianni *et. al* [14] the authors simplify the problem by not requiring any hydraulic modeling of the water distribution system, focusing only on the topology of the distribution network,

Berman, O., Larson, R. C., & Fouska, N. (1992). Optimal location of discretionary service facilities. *Transportation Science*, *26*(3), 201-211.

Berman, O., Bertsimas, D., & Larson, R. C. (1995). Locating discretionary service facilities, II: maximizing market size, minimizing inconvenience. *Operations Research*, *43*(4), 623-632.

***In order to make the paper more readable and to follow the whole procedure, I suggest to provide a clear general flow-chart of the framework you proposed;***

**Response:**

Exhibit 2 of the paper provides the description of the algorithm. We presented this flow chart in table form to give more details on each step of the algorithm.

***Have you assumed that only a contamination at time can start? Could you discuss all the assumptions behind your method?***

**Response:**

The setup of our problem consists of a set of sensors that monitor the state of the sewage network in real-time. When a sensor detects a surge of contamination from a sewage pipe, a manual sampling process is initiated to find the main source of the contamination. Consecutive sewage samples are taken and analyzed until the source manhole is identified. We now explain at the end of Section 2.

As mentioned earlier, in Larson et al. (2020) we provide two algorithms: one for finding the single source of contamination and one for finding the hot spot (the neighborhood with the highest level of contamination). It was shown in Larson et al. (2020) that the second algorithm (finding the hot spot) is a simple extension of the first. We show in Figure 8 that we can solve our problem of placing a minimum number of sensors to find also the hot spot. Again, the extension is very simple and is explained now in a paragraph before Figure 4.

***Page 16: “It also allows analyzing the structural properties of the sensor location problem including robustness to perturbations in sensor locations”. Could you clarify this point and provide more explanations?***

**Response:**

A robust solution is one that allows deviations in the objective function w.r.t. minor perturbations in the sensor locations. We explain in the paper that our derivation of robustness is possible due to having a closed-form expression for the objective function. The robustness can be evaluated by perturbing the location of sensors and assessing the variations in the closed-form objection function.

***What Qab stand for? What does it represent?***

**Response:**

$Q_{ab}$ is defined in mathematical terms in Step 2 of Exhibit 2. Given $Q_{ab}=\log\left( m_{a} \right)p_{a}+\log\left( m_{b} \right)p_{b}$ it is the number of samples in the catchment areas of sensors a and b. The two terms of $Q_{ab}$ appear in the objective function of the mathematical model. By choosing the largest $Q_{ab}$ the algorithm seeks to gradually lower the objective function by choosing to alter the catchment areas of sensors a and b with the largest $Q_{ab}$.

***What is the perspective of the future work? I propose to consider the possibility of completing the discussion on possibilities and limitations of the use of such analysis in other networked systems and infrastructures;***

**Response:**

We have almost completely modified Section 7 by breaking it into two subsections related to the required R&D moving forward and extended domains of the model’s applications. In the first one we discuss the perspective of future work and in the second we consider the possible usage of our methods to other applications.

***Could you also discuss about the computational complexity of your procedure? The scalability and the time requested for the analysis? This is important for the application to real big-sized systems;***

**Response:**

We believe the complete analysis of complexity is outside the scope of this paper which has mainly focused on developing a promising approach to finding an infected manhole or a viral hot spot using sensors and manual samples and developing an efficient heuristic algorithm that is able to provide near optimal solutions with robust characteristics. Instead of providing the analytical complexity of the sensor location problem, our numerical experiments on the tributary search algorithm (that is a central part of the algorithm used in this paper) show that the expected number of samples required to find the an infected manhole or a hot spot in a network of size n is log_2 (n) which is also the complexity of the bisection algorithm.

***Another crucial point is the fact that there is no explicitly reference to the validation of the proposed approach through performance indices especially because it is not tested on real case study neither compared with methods already developed. I strongly recommend to improve this aspect;***

**Response:**

We refrained from providing a case study of a real network because it could limit our analysis to a specific problem setting. Instead, our algorithm demonstrated in Exhibit 1 provides a flow chart for developing randomly generated and realistic sewage networks of various sizes. This network generation algorithm enables testing a wide range of networks which better generalizes our results.

***It is not clear the reason why the Authors presented a real case study, reconstruct the sewer network with a procedure and then they applied the sensor placement algorithm to another network. Could you clarify these aspects? Furthermore, why didn't you work directly on the real topology of the case study? All these hypotheses about the system could strongly affect the results?***

**Response:**

Our motivation is to test the algorithm on a wide range of networks. Applying the algorithm on a single case study would limit us to the characteristics of that single network. Therefore, we developed a random tree generation procedure to produce a wide range of networks as our testbed. To calibrate the parameters of the random tree generation procedure, we used a realistic case study to avoid generating unrealistic trees. The case study is mainly used to estimate the branching probabilities in the random tree generation procedure.

***Reviewer #2: The paper “Placing sensors in sewer networks: A system to pinpoint new cases of Coronavirus” presents a method to simulate tree networks, that replicate real sewer pipeline networks, for determining the number of sewage testing required to trace it back to the source of the infection and it addresses the sensors location problem in order to minimize the number of manhole samples required to find the ‘source manhole’. Heuristics are developed to solve this non-linear optimization problem. The content of the paper is a topical issue that may offer points for further research and new operational paths. However, I have several issues (see Major comments below) with the paper and therefore I cannot recommend acceptance in present form. The English usage is not of publication quality and requires major improvement to facilitate reading fluency and comprehension. A few suggestions in this respect are listed in the minor comments below.***

**Response**

We have revised the writing style of the paper according to your recommendations. Please see the tracked changes version of the submitted manuscript.

**MAJOR COMMENTS:**

***- Page 2 line 9 – “We build on a new field, “Wastewater-Based Epidemiology” (WBE)”. Several strategies in analyzing wastewater to measure biological markers of microbial disease have already been developed and efforts have been made in the field of monitoring sewer systems. At page 28 you state that “the most advanced effort appears to be in Israel, where Ben-Gurion University of the Negev has signed with the Health Ministry to monitor and detect COVID-19 in sewage samples from 14 Israeli communities” and cite the article “Israeli tech to hunt for early evidence of coronavirus in sewer systems” The Jerusalem Post, October 27, 2020. Consider adding a section with a proper state of the art overview, where you consider the major contributions to the field.***

**Response:**

The literature review section is extended. In section 2.1 we discuss the operational challenges of sampling manholes, in section 2.2 we discuss the need for installing sensors, and in section 2.3 we discuss the state-of-the-art of the embedded sensors.

***There is no reference to the simulation software you used to test the proposed approach.***

**Response:**

We have not used an off-the-shelves simulation software. Our simulation approach for generating random trees is presented in Section 3.3. and Exhibit 1 where we introduce an algorithm for generating sewage networks of various sizes. We use this newly developed tree generation algorithm to test the performance of the sensor location problem in a wide range of networks.

***- Page 6 line 5 – “According to public works experts […] each test would require about one hour”. It is not clear which type of sewage testing you are referring to and which kind of testing methods are currently available to analyze manhole samples.***

**Response** Here we are assuming that the R&D on fast inexpensive tests (such as being carried out by Dr. Zhugen Yang, cited in the text) has been successful and can be utilized by non-experts in the field, while opening and sampling manholes. See

26. Yang, Z. et. al. Community Sewage Sensors for Monitoring Public Health. Environ. Sci. Technol. 2015, 49, 10, 5845–5846. Hui, Q., Pan, Y., Yang, Z. Paper-based devices for rapid diagnostics and testing sewage for early warning of COVID-19 outbreak. [Case Studies in Chemical and Environmental Engineering](https://www.sciencedirect.com/science/journal/26660164). Vol. 2, Sept. 2020. <https://doi.org/10.1016/j.cscee.2020.100064>
27. Mao K, Zhang H, Yang Z. can a paper-based device trace COVID-19 sources with wastewater-based epidemiology? Environ. Sci. Technol. 2020, 54, 3733−3735.

***- In section 3.2 you call “Q” the ordered list of new pipeline segments to be created, while in section 5.3 you define = log() + log(). Clarify in which way Q and are related or consider using different letters to indicate the two variables.***

**Response:**

Thank you for brining this inconsistency to our attention. We have denoted the ordered list as O instead of Q in section 3.

***- Page 9 line 5 – you make a reference to a figure (Figure 1), depicting a simple catchment zone in the interior of a city block, that is not present in the manuscript. Consider including it in the present work or delete the reference.***

**Response:**

Thanks for this comment. We have deleted the reference.

***- Page 19 – “the number of sensors given in (2) is based on two strong assumptions. First, it assumes all manholes have the same Bayesian probability, a useful assumption for obtaining an approximate number of sensors but may require fine-tuning when the Bayesian probabilities are not equal. Second, it assumes that the expected number of manholes can be used, ignoring the probability distribution.” These are strong assumptions that are a bit in contrast with what you stated in the previous sections.***

***How do these simplifying assumptions affect the results?***

**Response:**

Thank you for this comment. We have extended the numerical experiments by assessing the distribution of the number of required samples given that a fixed number of sensors are installed. Our computational experiments exhibit robustness in results with respect to changes in the Bayesian probabilities. Thus, the derived number of sensors (derived based on the assumption of equal Bayesian probabilities) is not too far from the optimal number of sensors. The analysis is provided in Figure 9 and its associated discussion.

**MINOR COMMENTS:**

***- Page 8 – “with the terminating street for vehicles often having a “stop sign”.” This sentence risks to confuse the reader, since you are still referring to sewage system tree networks. It is not clear what you mean by this sentence.***

**Response:**

Revised.

***- Page 8 – “the 4-intersection has three sewer pipes flowing into a fourth pipe” –> “the 4-segment intersection […]”.***

**Response:**

Revised.

***- Page 9 – “The infected “Newbie” individual need not be on the West-East street to the left of this intersection. That individual could be located just north or just south of the intersection.” Reformulate this sentence for a better understanding of its meaning.***

**Response:**

Revised to “The infected “Newbie” individual could be located in any of the three upstream directions from the source manhole: North, West or South.”

***- Page 7 – “three types of pipes, each is ascending diameter for increased flows” –> “******each has an ascending diameter for increasing flow conditions”.***

**Response:**

Revised to “The sewage tree network has at least three types of pipes, each with an ascending diameter for increasing flow conditions. The three pipe types are branch sewer, main sewer and trunk sewer.”

***- Page 7 line 2 – “between 200 to over 5,000 manholes” –> “between 200 and 5,000 manholes” or “from 200 to over 5,000 manholes”.***

**Response:**

Revised.

***- In general, the English format is a bit informal, try to avoid expressions like “with say” and rhetorical questions (see page 6 line 3).***

**Response:**

We have thoroughly revised the writing style of the paper according to your recommendations. Please see the tracked changes version of the submitted manuscript.
